# First record of non-mineralized cephalopod jaws and arm hooks from the latest Cretaceous of Eurytania, Greece

**DOI:** 10.1186/s13358-020-00210-y

**Published:** 2020-12-02

**Authors:** Christian Klug, Donald Davesne, Dirk Fuchs, Thodoris Argyriou

**Affiliations:** 1 Paläontologisches Institut Und Museum, Universität Zürich, Karl-Schmid-Strasse 4, 8006 Zurich, Switzerland; 2 Department of Earth Sciences, University of Oxford, Oxford, OX1 3AN UK; 3 UMR 7205 (MNHN - Sorbonne Université - CNRS - EPHE) Institut de Systématique, Évolution, Biodiversité, Muséum National D’Histoire Naturelle, 57 rue Cuvier, 75231 Paris Cedex 05, France; 4 SNSB-Bayerische Staatssammlung für Paläontologie und Geologie, Richard-Wagner-Straße. 10, 80333 Munich, Germany; 5 UMR 7207 (MNHN - Sorbonne Université - CNRS) Centre de Recherche en Paléontologie - Paris, Muséum National D’Histoire Naturelle, 57 rue Cuvier, 75231 Paris Cedex 05, France

**Keywords:** Cephalopoda, Ammonoidea, Desmoceratoidea, Coleoidea, Maastrichtian, Taphonomy

## Abstract

Due to the lower fossilization potential of chitin, non-mineralized cephalopod jaws and arm hooks are much more rarely preserved as fossils than the calcitic lower jaws of ammonites or the calcitized jaw apparatuses of nautilids. Here, we report such non-mineralized fossil jaws and arm hooks from pelagic marly limestones of continental Greece. Two of the specimens lie on the same slab and are assigned to the Ammonitina; they represent upper jaws of the aptychus type, which is corroborated by finds of aptychi. Additionally, one intermediate type and one anaptychus type are documented here. The morphology of all ammonite jaws suggest a desmoceratoid affinity. The other jaws are identified as coleoid jaws. They share the overall U-shape and proportions of the outer and inner lamellae with Jurassic lower jaws of *Trachyteuthis* (Teudopseina). We also document the first belemnoid arm hooks from the Tethyan Maastrichtian. The fossils described here document the presence of a typical Mesozoic cephalopod assemblage until the end of the Cretaceous in the eastern Tethys.

## Introduction

Fossil cephalopods are mainly known from preserved mineralized parts such as aragonitic phragmocones (e.g., nautilids: Tajika et al. 2020; ammonoids: Hoffmann et al. 2019, coleoids: Klug et al. 2016a, b, 2019; Iba et al. 2012; Wani et al. 2018; Hoffmann and Stevens 2020), calcitic jaws (e.g., nautilids: Saunders et al. 1978; Klug 2001; ammonites: Lehmann 1972; Morton & Nixon 1987; Engeser and Keupp 2002; Keupp and Mitta 2015; Tanabe et al. 2015), and calcitic rostra (e.g., belemnites; Hoffmann et al. 2016, 2019; Hoffmann and Stevens 2020; Iba et al. 2012, 2014). While soft parts are rarely preserved (Klug et al. 2015, 2019; Donovan and Fuchs 2016; Clements et al. 2016), originally chitinous body parts such as jaws, arm hooks, and radulae are occasionally found (Matern 1931; Mapes 1987; Fuchs 2006a; Landman et al. 2010; Kruta et al. 2011, 2020; Klug et al. 2005, 2010a, 2016a, b, 2017, 2020; Keupp et al. 2016; Fuchs and Hoffmann 2017; Mitta et al. 2018). Naturally, such discoveries add important anatomical information to improve our understanding of cephalopod evolution (Kröger et al. 2011; Klug et al. 2019).

For the preservation of chitinous structures, special taphonomic conditions are required (Allison 1988; Briggs and Wilby 1996; Clements et al. 2016). As far as coleoid jaws (or beaks or mandibles) and ammonoid upper jaws are concerned, these occur sometimes in black shales and platy limestones of conservation deposits (Konservat-Lagerstätten). Such preservation was documented from, e.g., the Devonian Hangenberg Black Shale in Morocco (Klug et al. 2016a, b), Carboniferous deposits of Bear Gulch in the USA (Landman et al. 2010; Klug et al. 2019; Mapes et al. 2019), the Jurassic Posidonia Slate, the platy limestones of Nusplingen and the Solnhofen-Eichstätt region of Germany (Klug et al. 2010a, 2015, 2016a, b, in press; Jenny et al. 2019), as well as the Late Cretaceous of Lebanon (Fuchs 2006a, b; Fuchs and Larson 2011a, b; Jattiot et al. 2015).

Here, we describe cephalopod jaws from thin-bedded marly limestones of late Maastrichtian age of the Pindos Unit, Eurytania, continental Greece. These sediments yielded rich fossil fish assemblages (Koch and Nikolaus 1969; Argyriou and Davesne in review). The cephalopod remains were discovered in the course of recent field work. They are documented and interpreted for the first time in this study. In addition, we describe cephalopod arm hooks from the Tethyan Realm of Maastrichtian age for the first time.

## Material

**Fig. 1:**
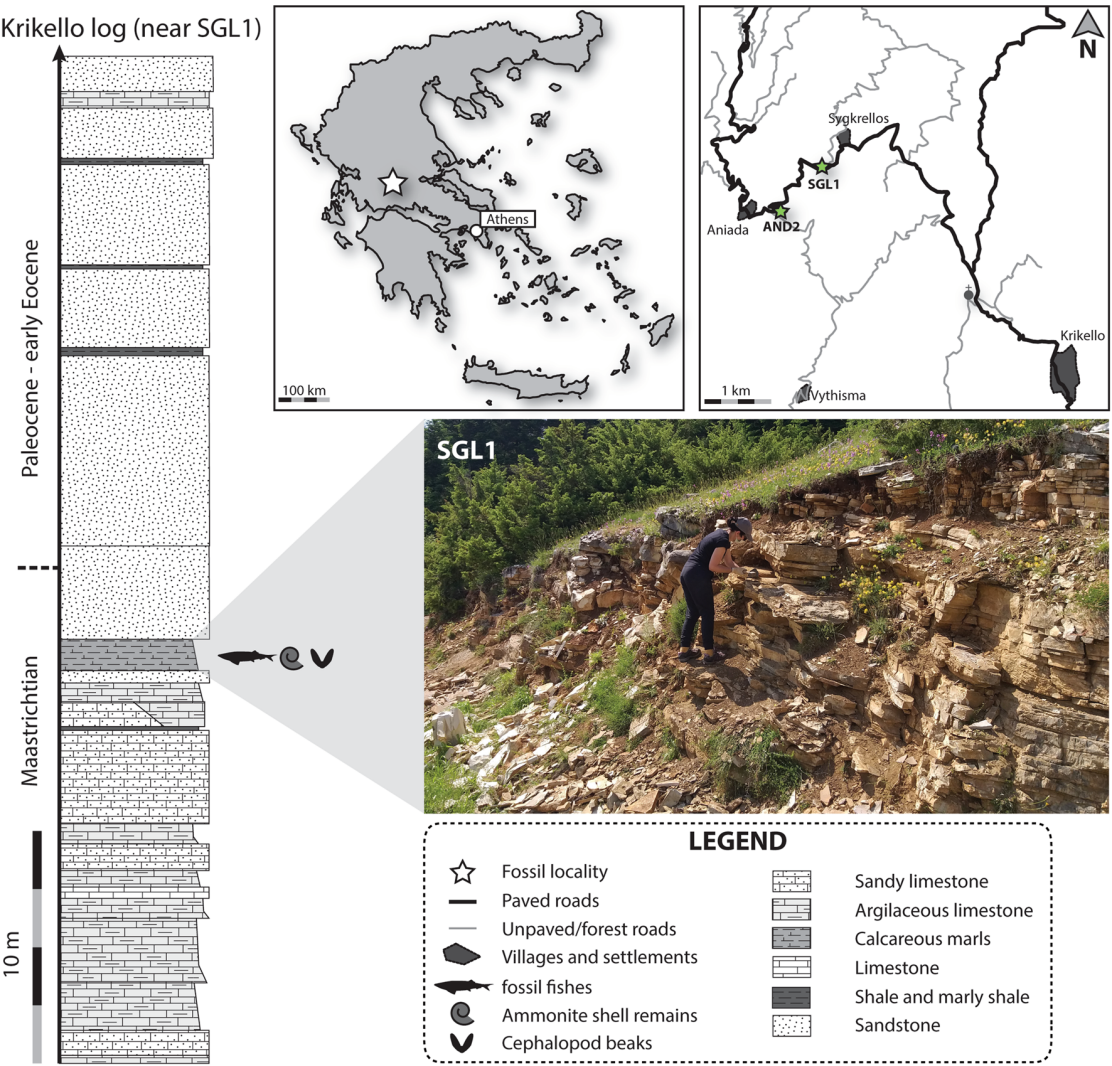
Location and geological information for the sampling sites SGL1 in Eurytania, continental Greece. AND2 corresponds to the same stratigraphic horizon as SGL1. Stratigraphic log sensu Koch and Nikolaus (1969), with the Cretaceous–Paleogene boundary arbitrarily moved higher, to be contained within the lower flysch deposits sensu Fleury (1980)

All cephalopod fossils are stored in the Museum of Geology and Palaeontology of the National and Kapodistrian University of Athens (AMPG). They were collected by TA and DD from Pindos Unit exposures in the newly discovered locality SGL1 near the village Sygkrellos, Eurytania, continental Greece, with an additional ammonite shell coming from locality AND2, near Aniada (Fig. 1). Locality SGL1 corresponds to a roadcut where the fossiliferous layers are exposed almost horizontally, at the road from Aniada to Sygkrellos. The material is usually preserved in slabs as part and counterpart. Catalogue numbers are as follows: AMPG_SGL1_4a,b, Ammonitinae ‘jaws’; AMPG_SGL1_22a,b, coleoid ‘jaws’; AMPG_SGL1_34, coleoid ‘jaws’; AMPG_SGL1_35, coleoid ‘jaws’; AMPG_SGL1_36, ?*Hauericeras* conch; AMPG_SGL1_40, ammonite aptychus; AMPG_AND2_1, desmoceratoid ammonite conch. To save space, we omit the prefix AMPG when referring to the specimens below.

### Geological setting and taphonomy

The fossils were found in late Maastrichtian marly limestone horizons of Pindos Unit, in Eurytania, continental Greece (Fig. 1). The Pindos Unit is a largely pelagic sedimentary succession initially associated with the Gondwanan oceanic margin of the greater ocean of Tethys, which later drifted and accreted—during the Maastrichtian–Danian—to the Eurasian continental margin (Papanikolaou 2013). Late Cretaceous lithofacies are dominated by pelagic platy limestones with occasional chert occurrences; the latter becoming rarer towards younger layers (Koch and Nikolaus 1969; Fleury 1980). The thin-bedded, grey–beige marly limestone horizons that yielded the fossils examined herein correspond to transitional facies between Mesozoic pelagic carbonate-dominated sediments and sandstone facies associated with the clastic, flysch deposits of latest Maastrichtian-Paleogene age (Koch and Nikolaus 1969; Fleury 1980; Papanikolaou 2013). Due to their complex geodynamic history (Papanikolaou 2013), the sediments of Pindos Unit in continental Greece have undergone severe tectonic deformation, and are nowadays found as heavily faulted and folded tectonic nappe series, each preserving incomplete sections of the original sedimentary succession (Koch and Nikolaus 1969; Fleury 1980; Papanikolaou 2013).

Although these fossiliferous, transitional marly limestones were initially thought to span the K–Pg boundary (Koch and Nikolaus 1969), their age for the region of Eurytania was later revised to the late Maastrichtian on the basis of globotruncanid planktonic foraminifers (see Fleury 1980 for more details). The overlying base of the flysch was biostratigraphically dated to the latest Maastrichtian, although the actual K–Pg boundary is probably situated in the first tens of metres of the sandy flysch facies and has not yet been pinpointed (Fleury 1980). Vertebrate fossils mostly belonging to enchodontoid and ichthyotringoid aulopiform teleosts (Koch and Nikolaus 1969; Argyriou and Davesne in review) are common in the same horizons that yielded the cephalopod remains described in this work.

## Results

Here, we employ the jaw terminology used by, e.g., Clarke (1962, 1986), Clarke and Maddock (1988), Klug et al. (2010b), Nixon (2015), and Tanabe et al. (2017) for the description of our jaw material. The arm hook terminology and morphometry follow Lehmann et al. (2011) and Fuchs and Hoffmann (2017). Primarily calcitic materials retained the original composition. Aragonitic conchs are dissolved and thus, ammonites are preserved as strongly flattened internal moulds, sometimes with phosphatized siphuncles. The cephalopod jaw elements that originally had a chitinous composition are preserved in a black or dark brown material. Superficially, this resembles coal or gagate/jet, but as demonstrated by Tanabe et al. (2019) for jaws from Japan, the black material might be apatite. The arm hooks are preserved as internal moulds, at least on the exposed side.

### Ammonite remains

In total, we could assign four jaw remains to ammonoids. Two are upper jaws and two are lower jaws. Both upper jaws are on the same slab, hence, there are only three specimen numbers. There are also some ammonite conchs, which could be determined only with great reservation because of their very poor steinkern preservation (strongly resembling the Solnhofen and Eichstätt mode of preservation; see, e.g., Schweigert 2009; Mapes et al. 2019: fig. 6).

**Fig. 2:**
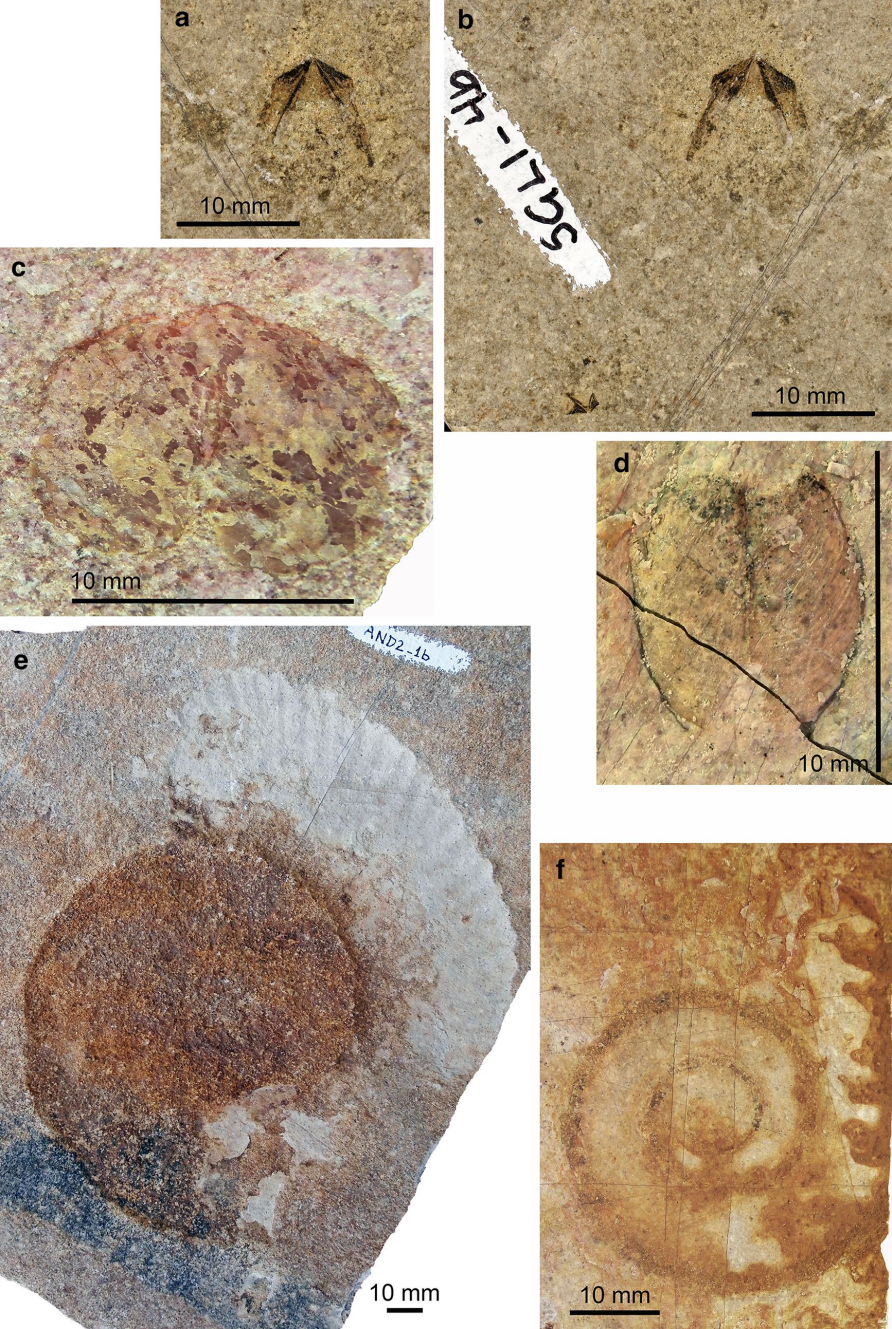
Ammonite remains from the Maastrichtian of continental Greece. **a**, **b** Slab and counterslab showing the dark remains of ammonitin upper jaws; SGL1_4b, a; note the small jaw on the bottom left in **b**. **c** Intermediate type of lower jaw, with both valves; SGL1_38. **d** ?*Lissaptychus* sp., with both valves; SGL1_40. **e** Poorly preserved ammonite conch (AND2_1) of a desmoceratoid, possibly of ?*Kitchinites* sp.; the body chamber is thicker than the phragmocone, which is heavily recrystallized. **f** ?*Hauericeras* sp.; SGL1_36; note the evolute conch lacking ornament and the biconcave aperture; the siphuncular tube of the last two whorls of the phragmocone are preserved. The brownish lobes on the right are calcitic dendrites, i.e. secondary formations linked to weathering and small-scale karstification. B, ammonite lower aptychus-type jaw, probably *Striaptychus*

**Fig. 3:**
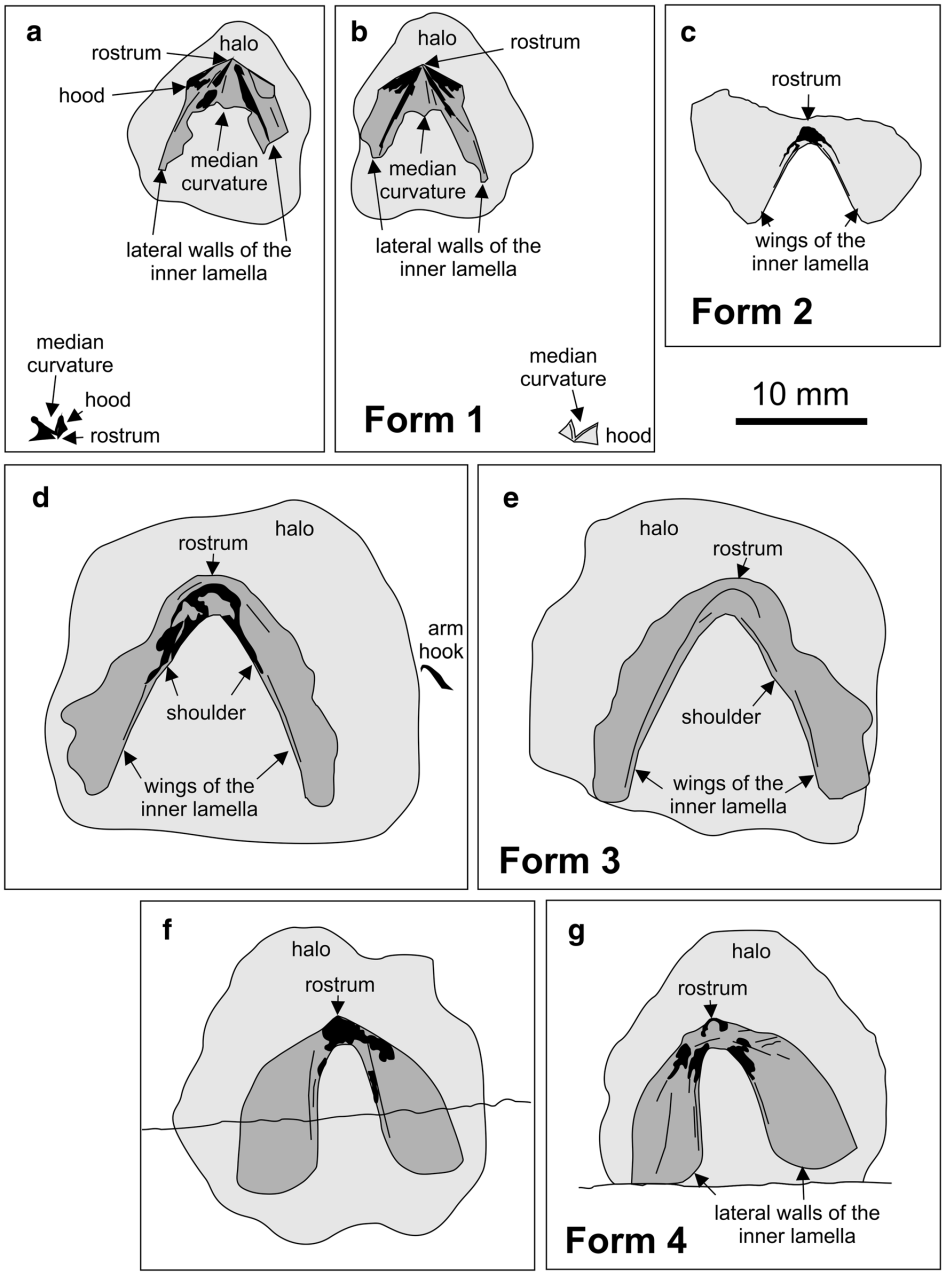
Some cephalopod jaws from the Maastrichtian of continental Greece. Note the halos (light grey) around all specimens. Black areas indicate the presence of sclerotized sheets. Middle grey areas indicate surfaces where the dark coating was very thin or broke off. **a**, **b** Slab and counterslab showing the apatitic remains of ammonitin upper jaws; SGL1_4b,a. **c** Lower jaw of a cephalopod, maybe an ammonite anaptychus, remotely resembling the lower jaw of *Vampyroteuthis*; SGL1_34. **d**, **e** Lower jaw of a teudopsein coleoid; SGL1_22a,b. **f**,** g** Lower jaw of a teudopsein coleoid; SGL1_35a,b

Specimen SGL1_4a and b are a small slab and counterslab of platy limestone, which display two quite similar jaws of different size (Figs. 2a, b, 3a, b). The remains of the larger jaw measure 8.5 mm in length and 10.4 mm in width. It is surrounded by a subcircular slightly yellowish halo with a diameter of about 15 mm. On both slabs, most of the outer and inner lamellae are visible as surfaces resembling coal or gagate (jet) due to its finely cracked surface and black shine (we did not analyse the material; following Tanabe et al. 2019, it might be apatite). The lateral walls of the inner lamellae are 10 mm long and about 1.8 mm wide. The width was likely reduced by compaction and was higher in the undistorted state. The inner edges of the lateral walls form an angle of about 40° and the outer edges form an angle of about 80°. The inner edges of the lateral walls are demarcated by a 6.5-mm-long dark line of thicker black material, which is a bit wider anteriorly (ca. 0.8 mm). The outer lamella and thus the hood delimit a triangle with a pointed rostrum that forms and angle of 125°. Posteriorly, its outer margins swing back into the lateral walls of the inner lamella. Towards the plane of symmetry, the hood displays a shallow median curvature. The thickness of the fossil remains of the hood varies. In two triangular fields at the outer anterior margins of the hood, the surfaces of the jaw form symmetrically arranged triangles with an angle of about 30°. The more strongly sclerotized rostrum reflects the thicker and more resistant chitin required to process prey items.

The smaller jaw has a very similar shape but displays less morphological detail due to its smaller size and proportionally thinner remains. It is about 3 mm wide and 2.2 mm long. The lateral walls of the inner lamellae are poorly preserved posteriorly. Mainly the more strongly sclerotized hood is discernible. The outer margins of the hood surround an angle of nearly 140°. These margins are gently curved, suggesting a formerly pointed rostrum. Two pairs of symmetrically arranged lines originate at the tip of the rostrum and run posteriorly. These lines form angles of 50° and 90°, respectively. The more distinct inner lines likely correspond tom the dorsolateral edges of the hood. The fainter outer lines might represent the connection between hood and inner lamella. The posterior edge of the surface displays a deep median sinus (median curvature). It is not clear how much of the inner and outer lamella are missing. Also, the lateral parts show deep indentations, which probably represent taphonomic artefacts due to thinner chitin.

The third jaw is specimen SGL_1_38 (Fig. 2c). Due to its bivalved nature, its bilateral symmetry, and its calcitic preservation, we interpret it as a broad lower jaw of an ammonite that preserves both valves. Each valve is 9.5 mm long and 6 mm wide. A keel is vaguely visible. The surface is so altered that no ornamentation is discernible. The overall shape allows two interpretations, namely ?*Striaptychus* sp. (sensu Trauth 1927, “Aptychenstudien II”) or a desmoceratoid jaw of the intermediate type. Examples for this type were illustrated by, e.g., Tanabe et al. (2015: fig. 10.5f) and Tanabe and Shigeta (2019) for the genus *Menuites*.

A second aptychus (SGL1_40; Fig. 2d) also preserves both valves but has a more slender morphology. A single valve is 8.5 mm long and 3.5 mm wide and slightly convex. A keel is not discernible, but with its fine concentric riblets, this specimen resembles some specimens of *Lissaptychus leptophyllus* on plate II of Trauth (1927, “Aptychenstudien II”: pl. II fig. 11–13). We thus tentatively assign it to ?*Lissaptychus* sp.

We also depict two ammonite conchs. As in other platy limestone conservation deposits, the conch is dissolved and the internal mould strongly flattened. Nevertheless, the siphuncle is occasionally preserved. The largest specimen (AND2_1; Fig. 2e) is a more or less complete conch with a diameter of 170 mm. While the phragmocone is completely flattened, covered by a calcite crust and preserving no ornamentation, the whitish body chamber (phosphoritic?) shows some weak relief. On a quarter whorl, it displays 22 prorsiradiate rounded ribs and 2 weakly visible constrictions. The whorl overlap is minimal, making the conch almost advolute. This character combination suggests an affinity to the Desmoceratoidea and, with great reservation, we suggest it might be a *Kitchinites* sp.

A second ammonite specimen (SGL1_36; Fig. 2f) has a conch diameter of 105 mm. The conch shows no ornamentation and the whorl overlap is low. The umbilical width amounts to ca. 67%. The last two whorls of the phragmocone display the siphuncle. The somewhat brighter body chamber is either very short (from the end of the siphuncle to the biconvex aperture is only a quarter whorl) or only partially preserved until a premature aperture or megastriae. There are two subparallel lines at the end of the discernible conch with the mentioned biconvex course. This conch morphology is reminiscent of ?*Hauericeras* sp., but naturally, this determination cannot be certain until better preserved material is found.

### Non-mineralized lower jaws (Figs. 3, 4)

**Fig. 4:**
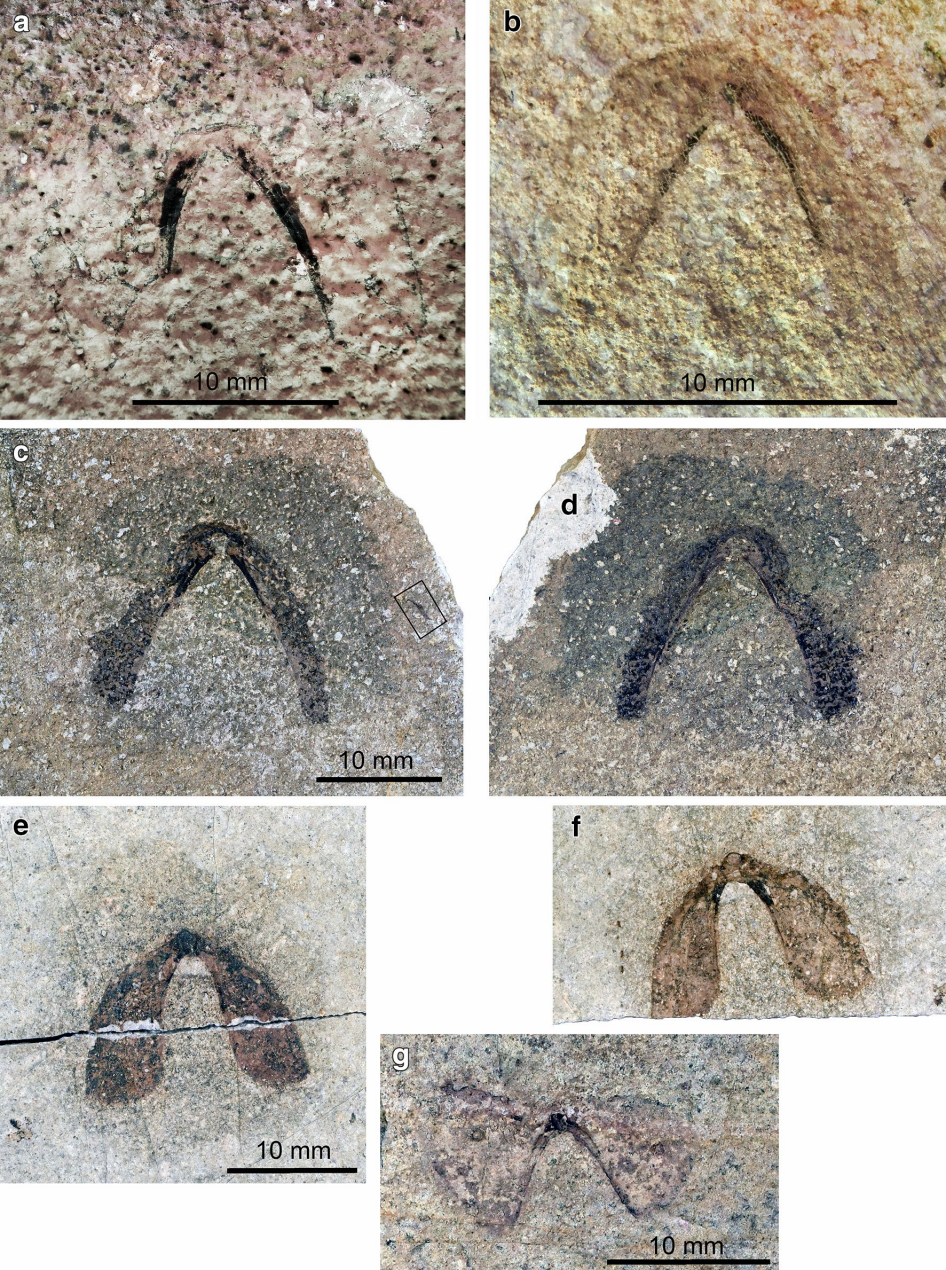
Cephalopod jaws from the Maastrichtian of continental Greece. Note the halos around all specimens. **a**, **b** Two poorly preserved lower jaws of a teudopsein coleoid. **a** SGL1_43. **b** SGL1_39. **c**, **d** Lower jaw of a teudopsein coleoid; SGL1_22a,b. **e**, **f** Lower jaws of a teudopsein coleoid; SGL1_35a,b. **g** Lower jaw of a cephalopod, probably of an ammonite, i.e. an anaptychus; SGL1_34

Specimen SGL1_22a and b are a small slab and counterslab of platy limestone bearing one jaw element (Figs. 3d, e, 4c, d). This jaw is 16 mm long and 19 mm wide. Like the other specimens, it is surrounded by a halo. The halo is slightly darker and has a diameter of about 25 mm. In this jaw element, the wings of the external lamella are much longer than the internal ones. The wings of the external lamella are about 17 mm long and about 4 mm wide with bluntly rounded posterior edges. Like the jaw described above, a fine but distinct line of black material demarcates the inner edge of the external lamella and the transition to the internal lamella. This dark line broadens anteriorly and grades into the up to 2 mm wide internal lamella. The middle of the internal lamella lacks the black coating on both slabs in an oval field that is 1.5 mm long and 1.2 mm wide and that tapers posteriorly. The same slab carries two coleoid arm hooks, which are described below.

Like the jaw described above, specimen SGL1_35a and b are also a small slab and counterslab of marly limestone bearing one jaw element (Figs. 3f, g, 4f, g). This jaw shares many morphological features with SGL1_22 (Fig. 4c, d) such as the long wings of the external lamella, the narrow ridges at the shoulders of the wings of the external lamella, which merge into the narrow internal lamella showing a light patch in the rostral part in the counterslab. The jaw is 17.3 mm wide and 13.6 mm long. The wings of the external lamella are much better preserved than in SGL1_22 showing a sharp outline. The wings curve and broaden posteriorly, reaching a maximum width of 6 mm. In the slab, the internal lamella is up to 2 mm wide and forms a small rostrum. The rostrum is strongly sclerotized on the slab, while on the counterslab, almost no black material remained, leaving a brighter oval patch of 2.3 mm length and 1.5 mm width. From the rostrum, the inner lamella quickly narrows posteriorly into the shoulder described above, which is about 0.5 mm wide in the middle of the external lamella, further narrowing posteriorly.

Specimen SGL1_43 (Fig. 4a) is a much less complete jaw. It is 18 mm wide and 11 mm long, but a few millimetres are missing both anteriorly and posteriorly. The lateral walls of the outer lamella are devoid of a carbonaceous coating and according to the visible imprint, they were already damaged prior to being embedded. The outer lamella still shows a well visible dark coating. Overall, the shape and proportions of this jaw resemble those of SGL1_22 and 35.

A fourth specimen (SGL1_39; Fig. 4b) also shares many aspects of the overall morphology, proportions and preservation of the previously described elements. The jaw is 8.5 mm long and 10.5 mm wide. It is complete, but the dark material is thinner than in SGL1_22 and 35. Like these jaws, it has a narrow outer lamella with a knob-like structure at the tip and broad lateral wings of the outer lamella.

The last jaw (Figs. 3c, 4h) described here looks quite different from the others and quite unlike most other coleoid jaws we know from the literature; accordingly, the assignment to a coleoid is uncertain. Specimen SGL1_34 lacks the counterslab. It measures 18.1 mm in width and 8.5 mm in length. It differs from the jaws described above in the much broader wings of the external lamella. By contrast, the inner lamella shows several similarities in its low width (as far as it is preserved and visible), the narrow shoulders, and the small, strongly sclerotized rostrum. The wings of the external lamella are triangular with a rounded posterior edge, an irregularly fractured anterior edge, and an anteriorly curved inner margin at the shoulders. Both wings of the external lamella are about 8.5 mm long. Near the posterior margin, the left wing is 10 mm broad whereas the right wing measures only 7 mm. The rugged anterior margin of both wings suggests that they might have been fused at this margin. The internal lamella is 0.6 mm wide at the shoulders and widens at the strongly sclerotized rostrum to a width of 1.2 mm.

### Arm hooks

**Fig. 5:**
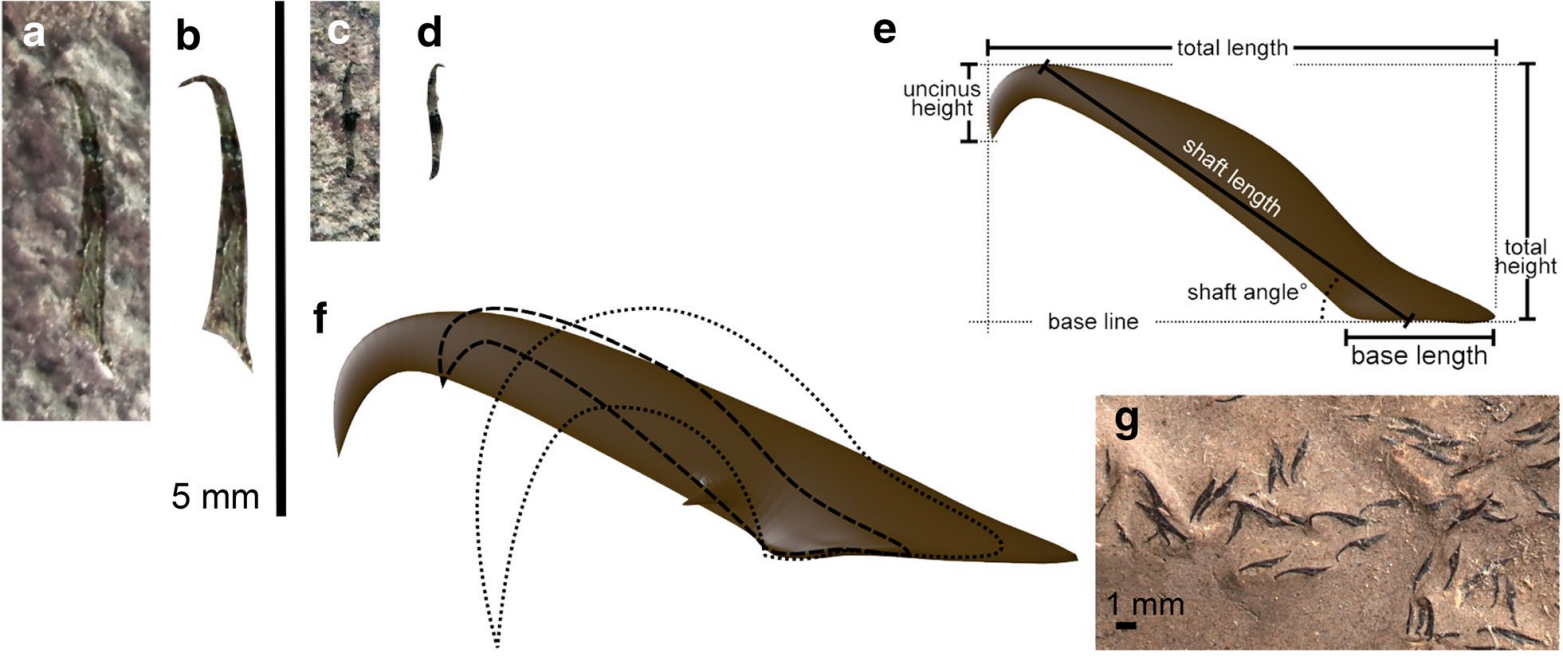
Belemnoid arm hooks from the Late Cretaceous of Greece and comparative morphology. **a**–**f** SGL1_22a, b, Maastrichtian; large vertical scale bar refers to **a** to **d**. **a** The larger arm hook on SGL1_22a. **b** Cut out from photo in **a** to better show the outline. **c** Second specimen from SGL1_22a. **d** Cut out from photo in **c** to better show the outline. **e** Reconstruction of second specimen (**c**, **d**) with terminology and measurements. **f** Comparison of various arm hooks: *Paraglycerites* sp. (Jurassic; brown shaded), “*Striatuncus*” *cretacicus* (Maastrichtian; dotted outline), and specimen SGL1_22a, b (Maastrichtian; dashed outline). **g** *Belemnoteuthis antiqua* (Callovian)

On the slab that contains the coleoid jaw SGL1_22, two small arm hooks are preserved. They are flattened and possibly slightly incomplete; hence, the measurements are not very accurate. The larger one of the hooks (Fig. 5a, b, f) has a total length of 2.9 mm with a base ca. 0.65 mm long. It has a long and slender shaft, which is weakly curved. The shaft is nearly 2.7 mm long while the uncinus measures only about 0.5 mm. Relative base length thus amounts to 32% and the relative uncinus length is 68% of hook length. The shaft angle is 35°. The ratio total height to total length is 0.52. The uncinus height in relation to the total height is 0.32.

Next to the halo, a 2.3-mm-long, poorly preserved arm hook is visible (Fig. 5c–e). On the counterslab, only its curved tip is discernible. The hook measures about 0.6 mm at its base. It is unclear whether this arm hook belonged to the same coleoid as the adjacent jaw; to clarify this, we have to await discoveries of more complete coleoid fossils from the Greek localities. The shaft is nearly 1.5 mm long while the uncinus measures only about 0.2 mm. Relative base length thus amounts to minimum 26% (probably more). The shaft angle is ca. 35°. These measurements are not very accurate because of the poor preservation.

## Discussion

**Fig. 6:**
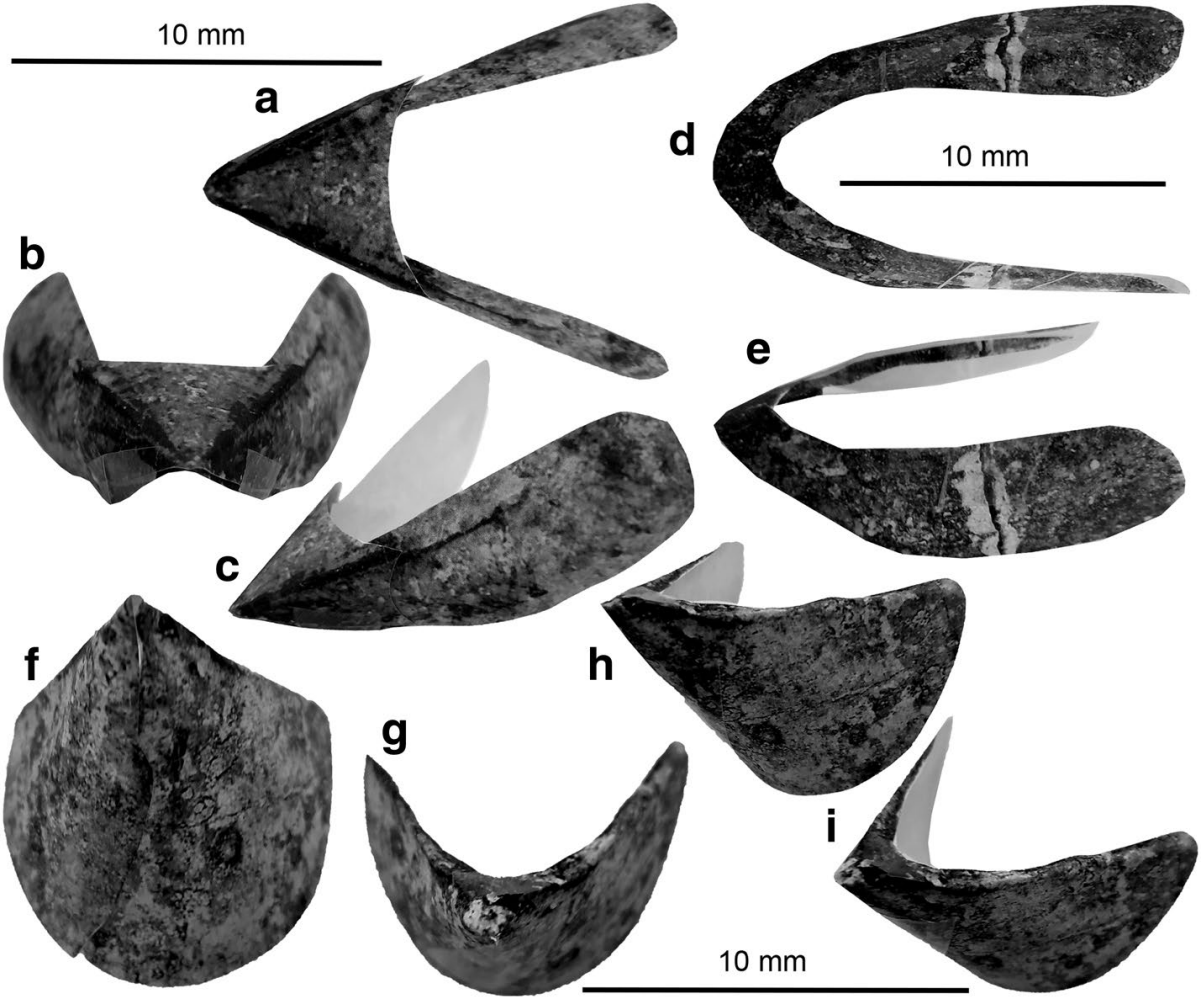
Paper reconstructions of some cephalopod jaws from the Maastrichtian of Greece. We printed the enlarged photos of the jaws, cut them out and taped them where they had ripped due to some taphonomic process (transport, compaction or else). **a**–**c** Ammonite upper jaw, maybe from a desmoceratoid, reconstructed after SGL1_4. **d**, **e** Teudopsein lower jaw, reconstructed after SGL1_35. **f** to **i** Lower jaw of the anaptychus type, maybe from a desmoceratoid, SGL1_34

The geometry and shape of the jaws on SGL1_4 resemble the upper jaws of ammonites (paper model in Fig. 6a–c). When comparing them to ammonite jaws, they strongly resemble upper jaws of the Late Cretaceous ammonite genera *Damesites*, *Menuites* and *Reesidites* (Tanabe and Landman 2002: fig. 2). A closer determination is probably impossible because the morphological detail is insufficient and in general, the upper jaws of ammonoids are rarely preserved and if they are, they are rarely complete and often in a poor condition.

Ammonite aptychi and anaptychi as well as some more enigmatic putative cephalopod jaws have been reported from several localities and sedimentary units in Greece, such as the Jurassic and Early Cretaceous of the Ionian Unit in Western Greece (Renz 1955, 1978; Bernoulli and Renz 1970), or the Early Cretaceous of Pindos Unit (Koch and Nikolaus 1969). However, to our knowledge, there have neither been previous mentions of cephalopod jaws from Maastrichtian horizons from the country, nor any mentions of this extremely rare type of chitin preservation. Here, we illustrate three different types of lower jaws (Fig. 2c, d). The smaller lower jaw SGL1_40 belongs possibly the aptychus genus ?*Lissaptychus* sp., while the slightly larger and much broader form SGL1_38 looks more like an intermediate type. The latter type is commonly assigned to desmoceratoids like *Menuites*. This is interesting since this coincides with our interpretation of the upper jaws.

Ammonoid conchs are very rare in the latest Maastrichtian strata of Pindos Unit in Eurytania and occur often in a flattened state with siphuncle preservation (Fig. 2f), which is known similarly from the platy limestones of southern Germany. This mode of preservation hampers species-level determination because of the lack of morphological detail. We note though that the ammonite genus *Gaudryceras* has been tentatively reported from the Maastrichtian of Eurytania (Renz 1955; Koch and Nikolaus 1969). The two specimens depicted in Fig. 2e, f were assigned to the desmoceratoid genera *Kitchinites* and *Hauericeras* with great reservation. Remarkably, this agrees with our determination of the upper and lower jaws. As long as in situ finds of buccal elements in ammonite body chambers are missing, we cannot make any certain assignments here.

The jaw specimens SGL1_22, 35, 39, and 43 probably belong to the same species (paper models in Fig. 6d, e). They resemble the lower jaws of Late Jurassic teudopseins like *Trachyteuthis* (Klug et al. 2005) in their long wings of the external lamella, the much narrower internal lamella with a very small rostrum. Also, the slightly wider anterior part of the anterior lamella around the rostrum is similar. A coleoid affinity is supported by the presence of an arm hook on SGL1_22. However, arm hooks are unknown from octobrachians.

SGL1_34 (paper model in Fig. 6g–i) looks quite different to SGL1_22 and SGL1_35. Presuming the external lamella was really fused anteriorly, this jaw would have somewhat resembled the lower jaw of the Recent *Vampyroteuthis infernalis* in its broad outer lamella (Clarke 1986, Klug et al. 2005: fig. 11A; Tanabe 2012: fig. 3:9a, b), but it differs in the shorter rostrum and the probably much shorter internal lamella. Taking the great similarity of SGL1_22 and SGL1_35 into account, we suggest that SGL1_34 really belonged to a different cephalopod group, possibly related to the modern genus *Vampyroteuthis*. Alternatively, it could be an ammonoid lower jaw of the anaptychus type (e.g., Dagys et al. 1989; Schweigert et al. 2016). Anaptychi share the broad outer lamella, the short inner lamella, and the taphonomic tendency to rupture radially with SGL1_34 (Schweigert et al. 2016). If this is correct, this would be a further indication for the presence of desmoceratoids in the Maastrichtian of northern Greece.

Compared to the Jurassic (Hart et al. 2016), arm hooks (both micro- and macro-onychites) are generally rare in the Cretaceous (Fuchs and Hoffmann 2017) and except very few records virtually unknown in the Late Cretaceous (e.g., Kulicki and Szaniawski 1972). Maastrichtian hooks have so far been reported only by Reich (2002) from the Rügen Chalk (Germany). The present arm hooks therefore represent the first records from the Maastrichtian Tethys. They were owned by ten-armed belemnoid coleoids rather than by eight-armed teudopseins (Fuchs and Hoffmann 2017). Their proportions deviate very faintly and thus belong to the same hook morphotype (possibly to the same individual or at least to the same species). Hook parameters suggest affinities to the paragenus *Paraglycerites* (Eisenack 1939)*.* The Maastrichtian hooks and mainly Jurassic *Paraglycerites* share a short base, a long shaft, and a low shaft angle. The uncinus height of the present hooks is unusually low by contrast to *Paraglycerites*, where the uncinus height occupies c. 50% of the total hook height. The Maastrichtian hooks therefore resemble those of the Jurassic orthogenera *Belemnotheutis* (Fuchs and Hoffmann 2017, figs. 4.1–2) and *Acanthoteuthis* (Schweigert 1999). However, belemnotheutins disappeared during the Early Cretaceous. The Maastrichtian hooks from the Rügen Chalk (“*Striatuncus*” *cretacicus* and *“Paraglycerites”* sp.) are fundamentally different in having uncini that reach or even exceed the base level (Reich 2002). Latest Maastrichtian belemnitids that roamed the northern Tethys are represented by only two genera of the family Belemnitellidae (*Belemnitella*, *Belemnella*). It therefore appears reasonable to attribute the two hooks to one of these rostrum-bearing belemnitids (see Riegraf 1996). Alternatively, the hooks might have belonged to the rostrum-less diplobelid *Conoteuthis* (Hauterivian—Maastrichtian), whose arms were probably also equipped with hooks (Fuchs et al. 2004).

Although our sample of cephalopod jaw comprises only a few jaw elements and two arm hooks, this association is typically Mesozoic. The two most important groups of Mesozoic cephalopods are still present, namely the ammonoids and belemnoids. Also, as far as other coleoids are concerned, the three jaws likely belonged to teudopseins. In the Jurassic and Cretaceous, belemnoids and octobrachians were the most important coleoid groups (e.g., Fuchs 2006a; Kröger et al. 2011; Clements et al. 2016). After the end-Cretaceous mass extinction, different groups of coleoids became more important such as the cirrate and incirrate octopodids and the crown decabrachians (i.e. excluding belemnoids and diplobelids). It is remarkable that many of the major Mesozoic cephalopod groups are indeed represented in this small faunule.

In addition to the above, these findings reveal the previously unknown potential of Maastrichtian rocks of Pindos Unit to preserve non-mineralized tissues, and provide rare insights into offshore Tethyan ecosystems of the Maastrichtian. Cephalopods lacking mineralized body parts, such as octobrachians, could have constituted prey items for the large numbers of predatory fishes (mostly enchodontoid and ichthyotringoid aulopiforms) that are abundant as fossils in the same horizons (Argyriou and Davesne in review). These rare cephalopod fossils add to an emerging picture of fully functional offshore ecosystems with conceived complex food chains at the very end of the Mesozoic Era, and just a couple of millions of years before the end-Cretaceous extinction.

## Conclusions

We described nine cephalopod jaws and two arm hooks from the latest Maastrichtian of continental Greece. Except the two calcitized aptychi, these fossils share a composition that was originally chitinous and their preservation in dark material (gagate-like, possibly apatite). They are preserved in marly platy limestones, which also yielded more or less articulated skeletons of teleost fishes. With some confidence, one aptychus (?*Lissaptychus*), one anaptychus and one of the intermediate type as well as two of the formerly chitinous upper jaws can be assigned to monomorph ammonites. Although a determination to family level is uncertain until such upper jaws are found within body chambers of determinable ammonites (only poorly preserved remains of *Kitchinites* and *Hauericeras* or similar genera), all lower and upper jaws as well as the ammonoid conchs suggest an affinity to the Desmoceratoidea.

The arm hooks and the other jaws belonged to coleoids. The belemnoid arm hooks (*Paraglycerites*-type) look like those of belemnoteuthins, while the lower jaws resemble those of Jurassic teudopseins (Vampyropoda) in four cases. Future field work will hopefully lead to the discovery of more complete coleoids with gladii and maybe arm crowns, which will help to test and refine our systematic interpretations.

Presuming these systematic assignments hold true, most of the major Mesozoic cephalopod groups would have been present in open Tethyan waters until shortly before the end-Cretaceous mass extinction. By contrast, remains of crown group octopodids and crown group decabrachians are missing; they are believed to have filled the vacant ecospace in the course of the subsequent rediversification.

## Data Availability

All specimens illustrated and described are stored at the Museum of Geology and Palaeontology of the National and Kapodistrian University of Athens, Greece.
